# Early psychosocial deprivation alters the refinement of neural dynamics across adolescence

**DOI:** 10.1073/pnas.2514979123

**Published:** 2026-02-02

**Authors:** Marta Andujar, Lucrezia Liuzzi, Daniel S. Pine, Charles A. Nelson, Charles H. Zeanah, Nathan A. Fox, Bruno B. Averbeck

**Affiliations:** ^a^Laboratory of Neuropsychology, National Institute of Mental Health, Bethesda, MD 20892; ^b^Emotion and Development Branch, National Institute of Mental Health, Bethesda, MD 20892; ^c^Laboratories of Cognitive Neuroscience, Division of Developmental Medicine, Boston Children’s Hospital, Boston, MD 02115; ^d^Department of Pediatrics, Harvard Medical School, Boston, MA 02115; ^e^Department of Psychiatry, Tulane University Health Sciences Center, New Orleans, LA 70112; ^f^Department of Human Development, University of Maryland, College Park, MD 20740

**Keywords:** neurodevelopment, pruning, dynamical systems

## Abstract

Adolescence is a crucial period for the refinement of neural circuits and cognitive skills. During this time, executive functions mature alongside pronounced structural changes in the brain, including reductions in synapse density. Computational models suggest that synaptic pruning enhances performance by stabilizing neural dynamics—deepening attractor basins and accelerating return to baseline after perturbation. Here, we tested these predictions using EEG data from the Bucharest Early Intervention Project, a randomized controlled trial of foster care as an alternative to institutionalized care. Trial-by-trial EEG residuals during a Flanker task were modeled as a linear dynamical system, and changes in eigenvalue magnitude were tracked across adolescence. Across all groups, eigenvalues decreased with age, consistent with maturation of recurrent computations. However, children with histories of institutional care exhibited consistently higher eigenvalues, suggesting delayed or disrupted refinement of the neural dynamics. Importantly, higher eigenvalues were associated with poorer behavioral performance on the task, linking altered neural dynamics to altered executive functions. These findings demonstrate that early deprivation leaves a lasting imprint on the developmental trajectory of neural dynamics, affecting the maturation of brain systems underlying cognitive control.

## Significance

Adolescence is a period of major behavioral and neural change. Here, we use modeling to examine how the trajectory of recurrent computations supporting cognitive control develops across adolescence. Using data from the Bucharest Early Intervention Project, we assessed how early adversity affects this trajectory. We analyzed electroencephalography (EEG) activity during a Flanker task and modeled trial-by-trial dynamics as a linear dynamical system. We found that eigenvalue magnitude—reflecting the temporal persistence of neural dynamics—decreases with age and that early psychological deprivation alters this trajectory. These findings align with theoretical predictions linking excitatory synaptic pruning to changes in neural dynamics and highlight the lasting influence of early experience on adolescent network refinement.

Adolescence entails rapid behavioral and cognitive maturation. Along with greater autonomy and heightened social sensitivity, there are marked improvements in executive functions and increased neural efficiency. This greater efficiency manifests as more stable task-evoked processing and, consequently, reduced trial-to-trial variability in both behavioral responses and neural signals (1–3). Neuroanatomically, this period is defined by synaptic remodeling: Gray matter volume steadily declines while white matter and myelination increase. Histological studies show excitatory synapse density peaking in late childhood before dropping by up to 40% during adolescence in a region-specific, hierarchical sequence (4, 5). Importantly, EEG and MEG studies concurrently report a shift from low- to high-frequency oscillations and stronger, more reliable task-evoked responses (6, 7). These parallel timelines raise the possibility that synaptic pruning itself drives the observed electrophysiological refinements. A recent computational study (8) proposed a mechanistic link between pruning and changes in recurrent cortical computations. In this model, pruning of recurrent synapses led to enhanced performance on working memory and reinforcement learning tasks. Critically, it also increased the system’s resistance to perturbations, meaning that deviations of neural activity from the average trajectory decays back to the mean more rapidly.

This theoretical framework has found empirical support in a recent longitudinal EEG study in adolescents (9). Using a linear dynamical systems model fit to trial-by-trial residual activity, the authors showed a developmental increase in the speed with which neural activity returns to baseline after perturbation, consistent with the emergence of stronger attractor dynamics during adolescence. Together, these findings provide a bridge between computational theory and empirical evidence, suggesting that the normative trajectory of synaptic pruning shapes the maturation of neural dynamics, ultimately supporting more efficient and stable cognitive processing.

Importantly, while this trajectory has been experimentally validated in typical developmental contexts, it remains unclear whether and how early adverse experiences, such as childhood deprivation, alter the maturation of neural computations. Current evidence suggests that adverse childhood experiences—such as abuse and neglect during sensitive developmental periods—can disrupt normative neuroplastic processes, leading to long-lasting alterations in brain structure and function in several cognitive domains (10–13). For example, some studies found that children raised in institutions show alterations in white matter integrity when compared to never-institutionalized peers (14, 15).

Other studies have also provided evidence of reduced cortical thinning during early adolescence in children who experienced early deprivation (11, 16) or psychopathology (17). MRI-derived cortical thickness has been interpreted as an indirect marker of synaptic density, although recent work has highlighted a significant contribution of intracortical myelination to this measure (18). Rodent studies, which allow for direct quantification of synaptic density, have provided converging evidence that early-life adversity alters the typical trajectory of synaptogenesis and pruning, leading to persistent increases in spine density and disrupted maturation of cortical circuits (19–21). Taken together, these findings suggest that adequate early nurturing relationships are essential for the normative refinement of cortical circuits and their functional properties.

In the present study, we leveraged the randomized, longitudinal design of the Bucharest Early Intervention Project (22) to probe 1) whether adolescence is characterized by a progressive decrease in the temporal persistence of task-evoked EEG activity—reflecting synaptic pruning–driven sharpening of attractor dynamics—and 2) whether early psychosocial deprivation delays or attenuates this trajectory. We found that the temporal persistence of trial-to-trial neural variability decreased into early adulthood in our sample—consistent with the emergence of stronger attractor-like dynamics as predicted by computational models (8) and observed in longitudinal EEG studies (9). However, youth with early institutional histories showed a marked disruption of this trajectory, suggesting that early deprivation exerts an impact on the maturation of recurrent cortical computations. In summary, our results show that early psychosocial deprivation impedes the normal developmental trajectory of neural dynamics with persistent effects into early adulthood.

## Results

### Behavioral Results.

**Fig. 1.**
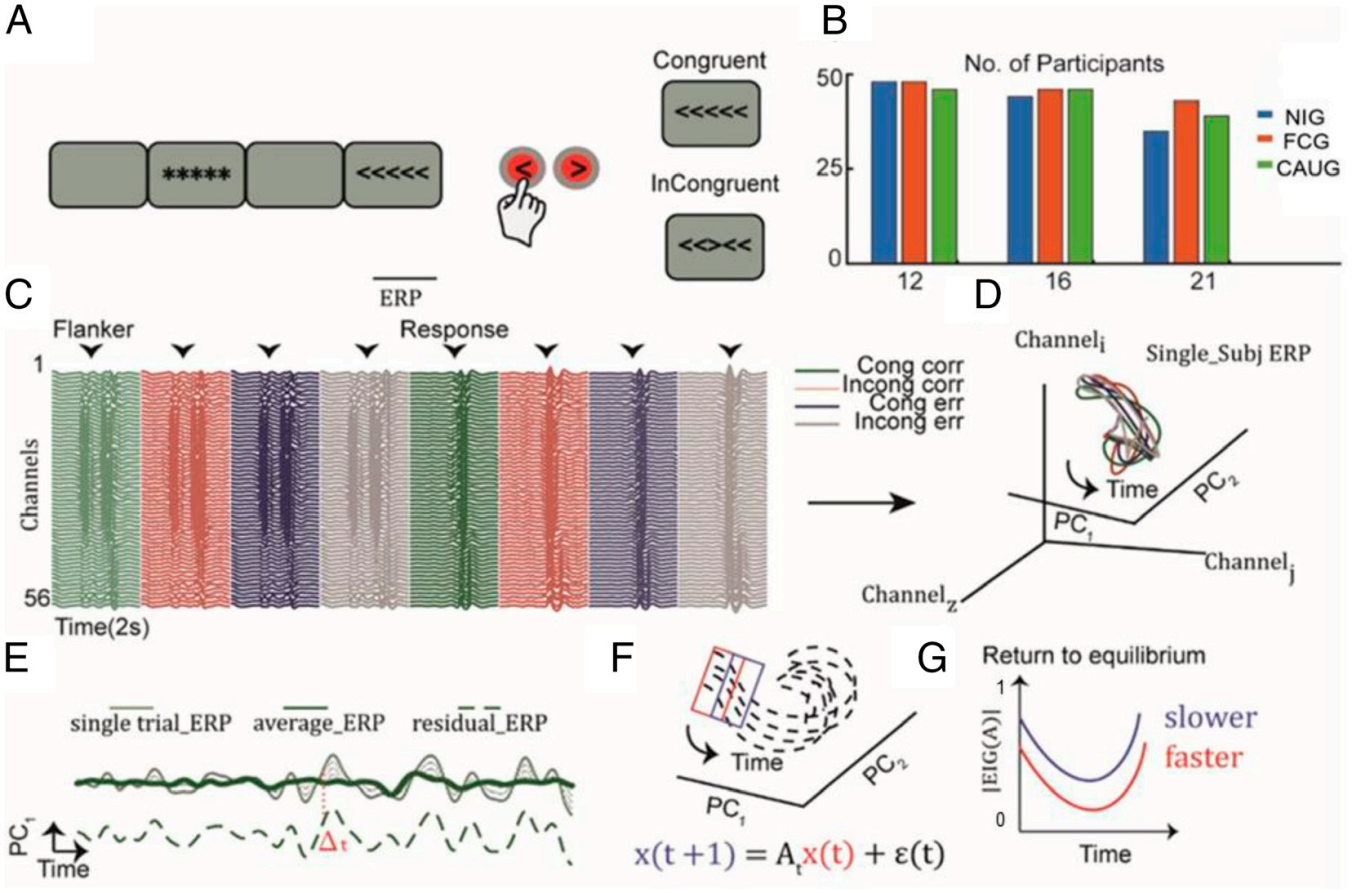
(*A*) Trial structure of the Eriksen Flanker task. (*B*) Number of participants at each age included in the study. (*C*) Time-concatenated ERP, averaged across all participants from 56 channels, aligned to two events of interest: the flanker stimulus and response onset. Each color represents the condition-averaged ERP (see legend) (*D*) Projection of the condition-averaged ERP from a single participant into the principal component (PC) space. The activity is aligned with the onset of the Flanker stimulus, and a 200-ms segment is displayed; data have been interpolated solely for representational purposes. (*E*) Example of condition-averaged ERP (thick line), single trial ERP (solid thin line), and residual ERP (dashed thin line) from a single participant projected onto the first PC. The residual activity is computed as the difference between the single-trial and the averaged activity over time in each component. (*F*) Cartoon: Residual activity (black dashed lines) in the 2D PC space and autoregressive model (see equation) used to predict the activity at the next time point (t + 1) based on the current one (t). (*G*) Eigenvalues of the autoregressive model as a proxy for network resistance to perturbations: Values closer to 1 reflect slower decay toward the mean state, while values near 0 indicate a faster return to equilibrium.

To assess developmental trajectories and the effects of early psychosocial deprivation on performance in the Flanker task—in which participants responded to the direction of a central target arrow flanked by congruent or incongruent distractors (*Materials and Methods*; Fig. 1*A*)—we examined accuracy, reaction time (RT), and the Balanced Integration Score (BIS). The BIS reflects a composite of speed and accuracy with equal weighting, quantifying overall performance such that higher values indicate faster and more accurate responding, whereas lower values reflect slower and less accurate performance.

Participants were divided into three groups differing in early caregiving experiences: the Never-Institutionalized Group (NIG), consisting of children who had never experienced institutional care; the Foster Care Group (FCG), comprising children who experienced early institutional care but were later placed in high-quality foster families; and the Care-As-Usual Group (CAUG), who remained in institutional care and thus experienced more prolonged psychosocial deprivation.

Behavioral performance was analyzed using linear mixed-effects models (LME) with Age (12, 16, and 21 y), Group (NIG, FCG, CAUG), and Condition (congruent vs. incongruent) as fixed factors (*Materials and Methods*).

**Fig. 2.**
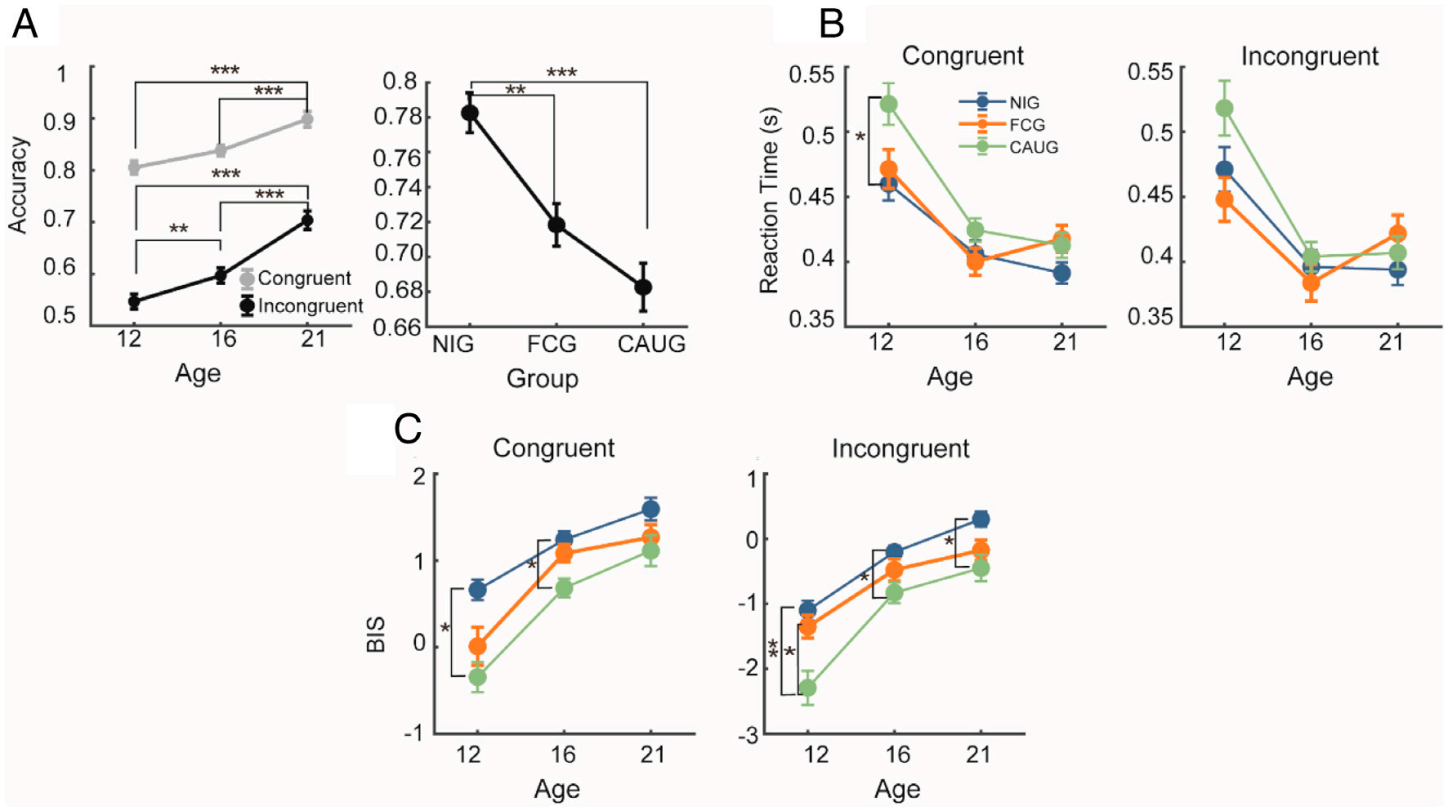
(*A*, *Left*) Two-way interaction (Age × Condition) on accuracy. Dots represent the group means (Congruent trials, gray; Incongruent trials, black); bars represent the SEM. (*Right*) Main effect of Group on overall accuracy derived from the same model, showing lower accuracy in FCG and CAUG compared to NIG. (*B*) Three-way interaction (Age × Group × Condition) on reaction times (RT). (*C*) Three-way interaction (Age × Group × Condition) on the balanced integration score (BIS). Same color code as in (*B*) (NIG = blue, FCG = orange, CAUG = green). In all panels, asterisks indicate significant post hoc pairwise contrasts from the mixed-effects model (Bonferroni-corrected; *P < 0.05; **P < 0.01; ***P < 0.001).

For accuracy, the model revealed significant main effects of Age (F (2, 121.38) = 34.09, *P* < 0.001), Group (F (2, 156.52) = 13.13, *P* < 0.001), and Condition (F (1, 163.14) = 524.75, *P* < 0.001), as well as an Age × Condition interaction (F (2, 143.56) = 5.78, *P* = 0.004). All other interactions were nonsignificant (all *P* > 0.05). Post hoc tests showed that accuracy increased with age in both conditions but more steeply for incongruent trials (Fig. 2*A*). In the congruent condition, accuracy differed between 12- and 21-y-olds (*P* < 0.001) and between 16- and 21-y-olds (*P* < 0.001), whereas 12- and 16-y-olds did not differ (*P* = 0.066). In the incongruent condition, accuracy improved across all age comparisons (all *P* < 0.01). NIG participants were more accurate than both FCG (*P* = 0.003) and CAUG (*P* < 0.001), who did not differ (*P* = 0.265).

For reaction times, the model revealed significant main effects of Age (F(2, 128.63) = 47.21, *P* < 0.001) and Condition (F(1, 143.91) = 463.29, *P* < 0.001), together with significant Age × Group (F(4, 128.23) = 3.34, *P* = 0.012), Age × Condition (F(2, 121.56) = 9.93, *P* < 0.001), Group × Condition (F(2, 143.89) = 3.63, *P* = 0.029), and Age × Group × Condition (F(4, 121.23) = 2.86, *P* = 0.026) interactions. Although modest in size, the three-way interaction indicated that group differences were confined to early adolescence (Fig. 2*B*): At age 12, CAUG participants responded more slowly than NIG in the congruent condition (*P* = 0.032 after correction), whereas no group effects were detected at older ages or in the incongruent condition (all corrected *P* > 0.05). Beyond this limited group-specific effect, RTs decreased with age more steeply in incongruent trials, consistent with greater developmental gains under higher conflict demands. RTs declined sharply from 12 to 16 y (*P* < 0.001) and then plateaued. Post hoc analysis of the Age × Group interaction did not reveal reliable between-group differences (all *P* > 0.05), indicating that developmental improvements in response speed followed a similar trajectory across groups.

For overall performance (BIS), the model revealed significant main effects of Age (F(2, 118.15) = 84.48, *P* < 0.001), Group (F(2, 161.01) = 12.59, *P* < 0.001), and Condition (F(1, 165.25) = 833.75, *P* < 0.001), together with significant Age × Condition (F(2, 141.62) = 3.22, *P* = 0.043) and Age × Group × Condition (F(4, 141.36) = 2.80, *P* = 0.028) interactions. Post hoc analyses indicated that group differences in performance were most pronounced under high control demands (Fig. 2*C*). At age 12, CAUG participants performed significantly worse than NIG across both conditions (congruent *P* = 0.004; incongruent *P* = 0.001) and worse than FCG in the incongruent condition (*P* = 0.016). At age 16, the NIG > CAUG difference persisted (congruent *P* = 0.001; incongruent *P* = 0.038), while FCG showed intermediate performance, not differing from either group (all *P* > 0.05). By age 21, only the NIG > CAUG contrast remained significant in the incongruent condition (*P* = 0.010).

Taken together, these findings highlight a consistent developmental pattern: Cognitive control becomes more accurate, faster, and more efficient with age, while early institutional deprivation leaves a residual performance cost that—although partly remediated by foster care—remains detectable across behavioral indices.

### Neural Results.

**Fig. 3.**
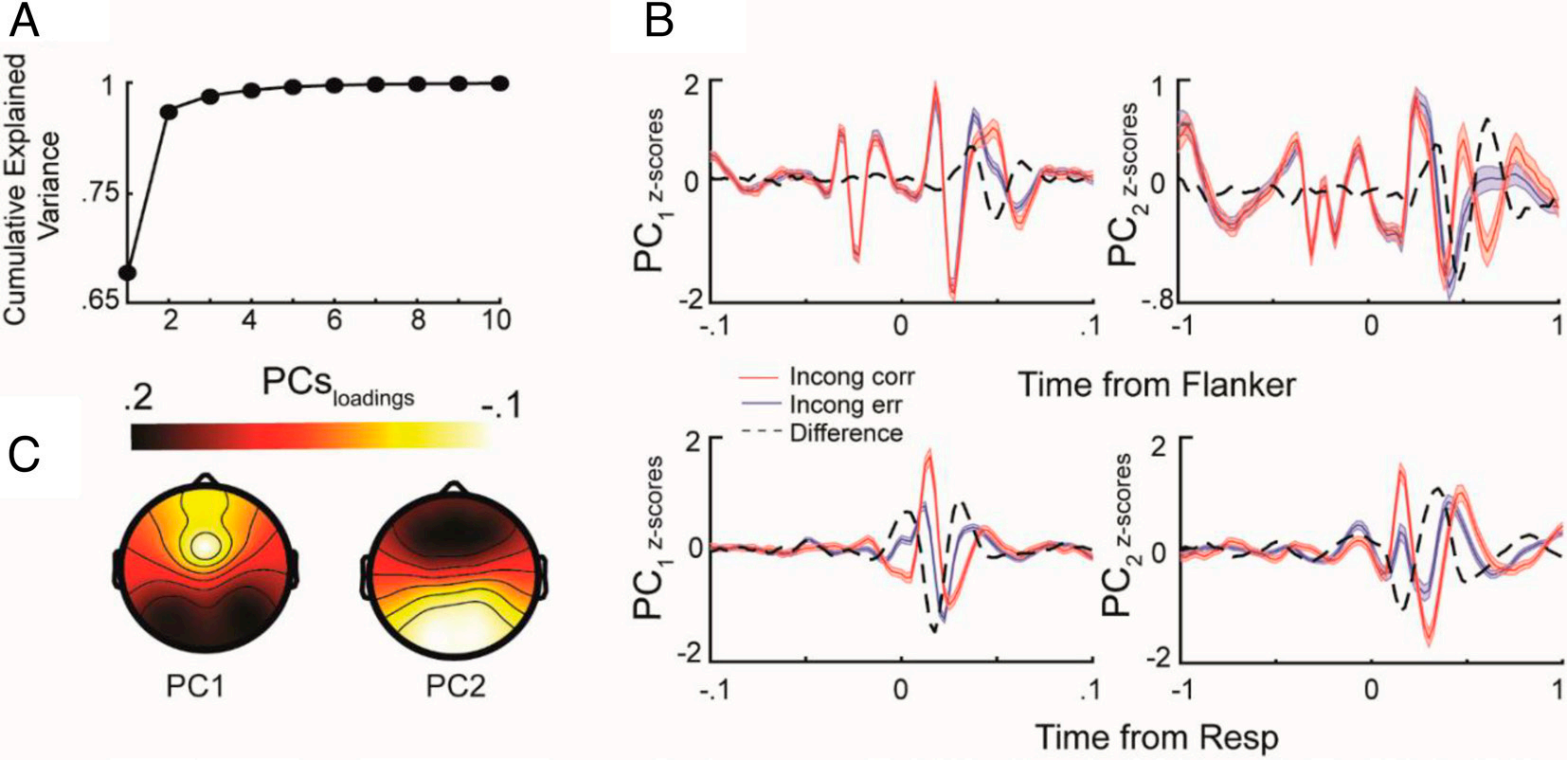
(*A*) Cumulative explained variance as a function of the number of principal components. (*B*, *Top*) EEG activity projected onto the first (*Left*) and second (*Right*) principal components, time-locked to flanker stimulus onset (*Top*) and response onset (*Bottom*), for two representative conditions (Incongruent correct trials in red, incongruent error trials in blue). Thick lines denote the across-participant and across-age group average, while shaded areas represent the SEM. The dashed black line represents the difference between conditions. (*C*) Topographical distribution of the weights for the first and second PC, which define the state space.

We next investigated 1) how the developmental transition from puberty to early adulthood influences the dynamics of the task-evoked EEG signal and 2) whether early deprivation experiences affect the developmental changes of the dynamics. To examine this, we characterized the dynamics of task-evoked neural activity by modeling the temporal evolution of residual neural activity in principal component space (Fig. 3). Residual activity captures how neural activity evolves after a perturbation (Fig. 1 *E*–*G*). The eigenvalues of the autoregressive model quantify this process, with larger eigenvalues indicating prolonged deviations and smaller eigenvalues reflecting a faster return to the mean trajectory.

**Fig. 4.**
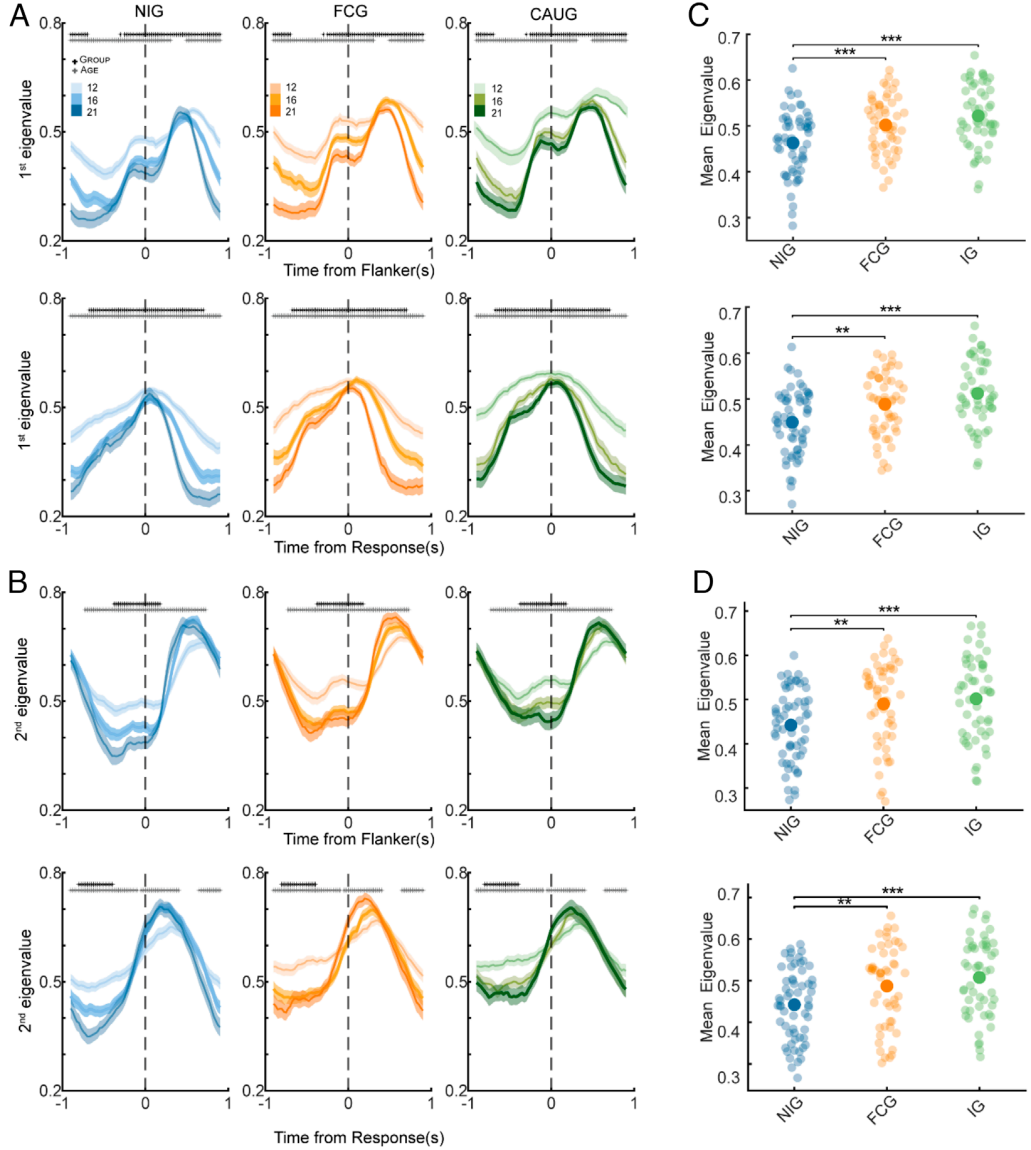
(*A*, *Top*) Temporal evolution of the first eigenvalue across ages (see color legend in *A*) and experimental groups (from left to right: NIG, FCG, and CAUG groups) during the flanker epoch. (*Bottom*) Same as in the *Top* panels but for the response-aligned epoch. (*B*) Same as in (*A*) but for the second eigenvalue. In all the panels, the colored thick line represents the average across participants and the shaded area represents the SEM. (*C*) 1st eigenvalue averaged within the longest time cluster showing a significant group effect (LME, *P* < 0.05). Larger opaque circles represent the mean for different groups (see legend) and error-bars. Each transparent dot represents the eigenvalue for one participant. Asterisks on the bars indicate significant differences between means (*P* < 0.05: *, *P* < 0.01: **, *P* < 0.001: *). (*D*) Same as in *C* but for the second eigenvalue.

Analysis of the first eigenvalue revealed significant main effects of both age and group, which persisted across nearly the entire flanker-aligned and response-aligned intervals (Fig. 4*A*). In contrast, the second eigenvalue (Fig. 4*B*) showed temporally specific effects. Age-related differences were relatively diffuse over time, whereas group differences were more temporally clustered. We conducted post hoc analysis on the eigenvalues averaged within the time windows where the linear mixed-effects (LME) model revealed significant effects. The first eigenvalue showed a consistent negative scaling with age (Fig. 4*A*). In the flanker-aligned epoch (−900 to 300 ms), age differences were significant between 12 and 16 y (*P* < 0.001), 12 and 21 y (*P* < 0.001), and 16 and 21 y (*P* < 0.001). A similar pattern was observed in the later window (500 to 900 ms; 12 vs. 21 y: *P* < 0.001; 16 vs. 21 y: *P* < 0.001). In the response-aligned epoch (−900 to 900 ms), the same trend emerged, with significant differences between 12 and 16 y (*P* < 0.001), 12 and 21 y (*P* < 0.001), and 16 and 21 y (*P* < 0.001). In contrast, the second eigenvalue exhibited a task-specific pattern: Before response onset, it decreased with age (flanker-aligned epoch: −725 to 725 ms; 12 vs. 16 y: *P* < 0.001; 12 vs. 21 y: *P* < 0.001; 16 vs. 21 y: *P* = 0.600), but upon movement onset, this trend reversed (response-aligned epoch: −50 to 375 ms; 12 vs. 16 y: *P* < 0.001; 12 vs. 21 y: *P* < 0.001; 16 vs. 21 y: *P* = 0.002), showing an age-related increase in eigenvalue magnitude (Fig. 4*B*)

When examining group effects, eigenvalues were consistently lower in the NIG group compared with both FCG and CAUG, which did not differ from each other.

For the first eigenvalue in the flanker-aligned epoch (Fig. 4*C*; −250 to 900 ms), NIG showed significantly lower values than FCG (*P* < 0.001) and CAUG (*P* < 0.001), while no difference emerged between FCG and CAUG (*P* = 0.480). A similar pattern was observed in the response-aligned epoch (−675 to 700 ms), where NIG again differed significantly from FCG (*P* = 0.001) and CAUG (*P* < 0.001), whereas FCG and CAUG did not differ (*P* = 0.110). The same pattern of differences was found for the second eigenvalue (Fig. 4*D*): NIG showed lower values than both FCG and CAUG, who did not differ from each other in either epoch (flanker-aligned epoch: −375 to 175 ms; NIG < FCG, *P* = 0.002; NIG < CAUG, *P* < 0.001; FCG vs. CAUG, *P* = 0.600; response-aligned epoch: −800 to −400 ms; NIG < FCG, *P* = 0.004; NIG < CAUG, *P* < 0.001; FCG vs. CAUG, *P* = 0.300).

In summary, we found that overall, the eigenvalues decreased with age. Notably, this age-related effect was not uniform across all task phases, hinting that the decay of residual activity may emerge at different rates depending on the brain area and cognitive operation being engaged (e.g., deliberation versus response monitoring, see *SI Appendix* for further discussion). In parallel, we found that children with a history of early-life deprivation exhibited consistently higher eigenvalues compared to their never-institutionalized peers, suggesting that early adversity may disrupt the typical tuning of recurrent computations with effects that extend into early adulthood.

We next examined whether eigenvalues were related to behavioral performance as indexed by the BIS. Because stronger attractor dynamics—reflected in lower eigenvalues in our framework—are associated with higher decision confidence and reduced susceptibility to distractors, (8, 23), we expected a negative association between eigenvalues and BIS across age and groups.

**Fig. 5.**
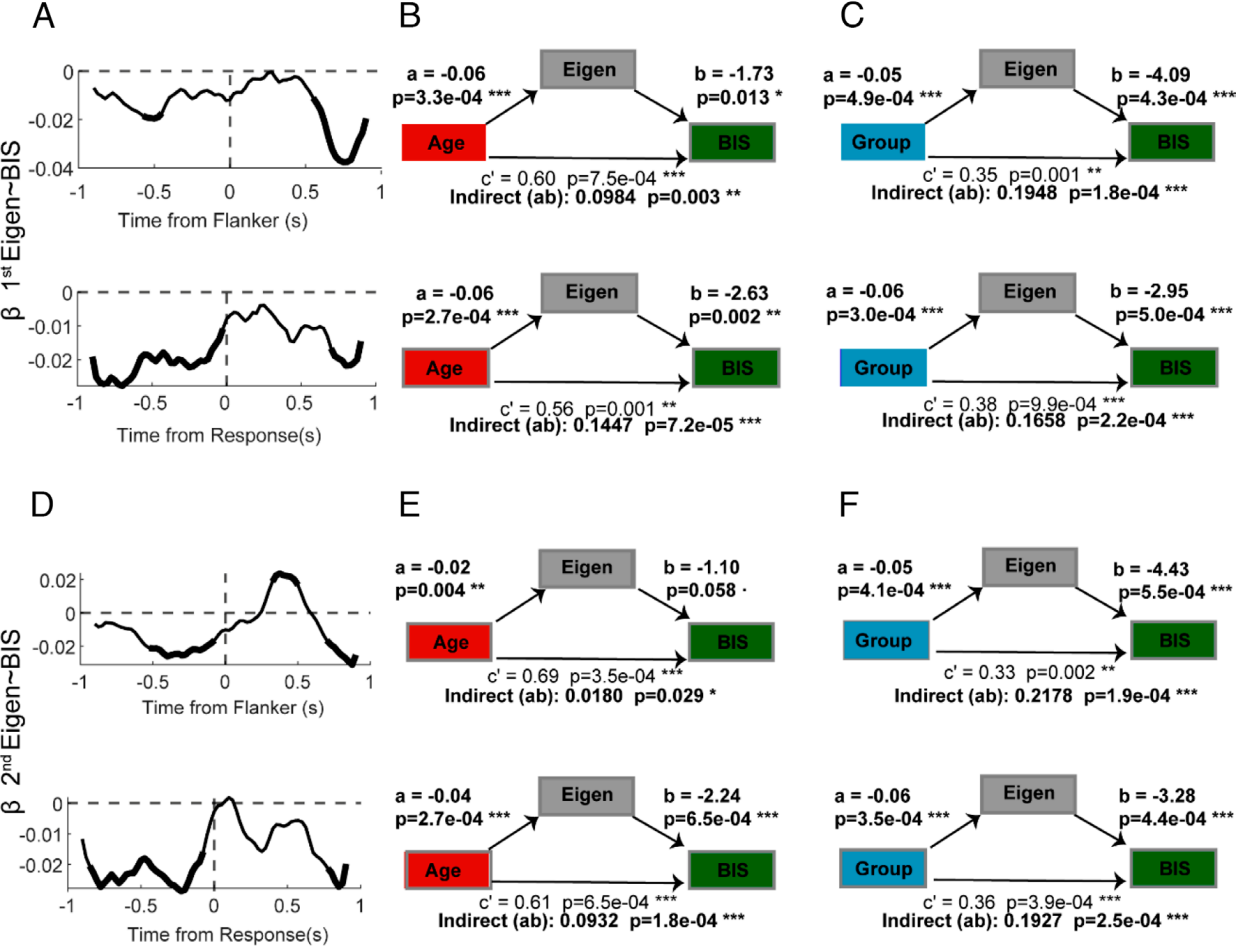
(*A*, *Left*) Linear effect of BIS on the first eigenvalue aligned to flanker (*Top*) and response (*Bottom*) onset. (*B*) Mediation analyses testing whether eigenvalues statistically mediated the effects of Age on task performance. Path coefficients indicate standardized effects: a = Age → eigenvalue, b = eigenvalue → BIS (controlling for predictor), c′ = direct effect of Age → BIS (controlling for eigenvalue), and ab = indirect effect (mediation). The *Top* panel shows results for the Flanker epoch, while the *Bottom* panel shows those for the Response epoch. (*C*) Same as in (*B*) but testing whether eigenvalues mediated the effect of Group on BIS. (*D*–*F*) Corresponding analyses for the second eigenvalue, following the same structure as panels (*A*–*C*). Significant effects are marked by asterisks (*P* < 0.001 = ***, *P* < 0.01 = **, *P* < 0.05 = *).

We found that both eigenvalues were significantly related to task performance. The first eigenvalue showed a negative association with BIS in both the Flanker and Response epoch (−575 ms to −475 ms, 550 ms to 850 ms; Response epoch: −900 ms to −20 ms, 725 ms to 900 ms; Fig. 5*A*), indicating that lower eigenvalues were linked to higher behavioral efficiency. Further, this relationship was modulated by age, emerging progressively from early to late adolescence: It was negligible at 12 y, became more negative at 16, and further strengthened by 21 y (*SI Appendix*, Fig. S1). Similarly, the second eigenvalue showed an overall negative association with BIS throughout the Flanker epoch (Fig. 5 *D*, *Top*), punctuated by a brief reversal between 300 and 500 ms after stimulus onset. This transient positivity likely reflects residual variability in the onset of response-related activity across participants, which is minimized when signals are response-locked rather than stimulus-locked. Indeed, in the Response epoch, the second eigenvalue again showed a clear negative association with BIS (−800 ms to −100 ms and 775 to 900 ms), paralleling the pattern observed for the first eigenvalue (Fig. 5 *D*, *Bottom*).

To assess whether eigenvalue dynamics played a mediating role in the relationship between behavior, development, and early experience, we performed mediation analyses linking i) age, eigenvalues, and BIS, and ii) institutionalization, eigenvalues, and BIS. Both analyses revealed significant indirect effects (a × b paths; Fig. 5 *B*, *C*, *E* and *F*), indicating that developmental and experiential influences on performance were partially transmitted through changes in eigenvalues. The direct effect (c′ path; Fig. 5 *B*, *C*, *E* and *F*) remained significant across components and epochs, consistent with partial mediation, suggesting that eigenvalues capture a key—though not exclusive—mechanism linking cortical recurrent dynamics to behavior.

## Discussion

In this study, we investigated how task-evoked EEG dynamics develop during adolescence, and whether childhood neglect alters the developmental trajectory of these neural processes. To this end, we fit a 2-dimensional linear dynamical system model to the residual EEG activity projected into a 2-D principal component space. We then computed the trial-time-dependent eigenvalues of the dynamics matrix and examined them in relation to participants’ age and history of deprived caregiving.

Longitudinal analyses revealed that eigenvalues, reflecting the rate at which residual activity returns to the mean trajectory, systematically decreased with age, consistent with the idea that synaptic pruning contributes to the maturation of recurrent cortical dynamics (8, 9). During adolescence, pruning removes weaker synapses, yielding a sparser yet more efficient recurrent architecture (24). By reducing diffuse connectivity and reinforcing stronger connections, this process likely reshapes cortical dynamics, creating deeper attractor basins around dynamical trajectories. These basins are stable patterns of population activity toward which the system naturally returns following a perturbation. Such deeper basins can stabilize task-relevant activity while allowing flexible transitions when inputs change. Computational models show that the balance and density of recurrent connections govern the depth and stability of attractor states: When excitation is strong, but connectivity remains sparse and balanced, networks sustain selective, persistent activity without runaway dynamics (25). Similarly, in artificial recurrent networks, removing weak weights during learning leads to networks with faster recovery from perturbations, consistent with smaller eigenvalues in the EEG data (8). Together, these principles suggest that the age-related decrease in eigenvalues observed here reflects the progressive consolidation of recurrent cortical dynamics, as pruning sculpts a sparser yet stronger network architecture that stabilizes task-relevant activity against perturbations. Although direct experimental evidence for this link remains limited, several converging findings from developmental electrophysiology support this interpretation.

First, ERP studies have consistently shown that neural responses supporting executive functions—such as error monitoring and conflict resolution—increase in amplitude and become more temporally precise across development, particularly during adolescence (7). Children, for example, exhibit delayed and sustained ERP responses to conflict compared to adults, reflecting immature attentional and conflict-monitoring systems (26, 27). Our results align with this trajectory: The systematic age-related reduction in eigenvalues suggests that noise-related variation in neural activity decays more rapidly to mean dynamics in older participants. Since eigenvalues quantify the temporal persistence of neural activity perturbations, this pattern indicates a developmental shift toward faster, more self-stabilizing neural processing. This functional refinement is likely driven by structural reorganization, in particular synaptic pruning, which eliminates diffuse connectivity and sharpens network architecture (28, 29).

While direct evidence linking ERP amplitude changes to cortical structure remains limited, some studies have demonstrated associations between ERP components and gray matter volume. In healthy adults, for instance, greater error-related negativity (ERN) amplitude has been negatively correlated with anterior cingulate cortex (ACC) volume, suggesting that more efficient error monitoring is supported by more refined, possibly pruned, cortical architecture (30).

Second, our findings may reflect a developmental reduction in low-pass filtering at the circuit level. Theoretical models suggest that dendritic and synaptic structures act as intrinsic low-pass filters, favoring slow activity while attenuating faster signals (31, 32). During adolescence, synaptic pruning and membrane maturation—particularly in associative cortices—may relax these constraints, permitting the propagation of faster, more temporally precise signals. This interpretation is consistent with resting-state EEG and MEG findings showing age-related decreases in low-frequency (e.g., θ, α) power and increases in higher-frequency (e.g., β, γ) power (7, 33), patterns linked to cortical thinning and reductions in gray matter volume (34). Consistently, our model—mathematically equivalent to a first-order low-pass filter—revealed a developmental reduction in eigenvalues, supporting the notion that adolescent cortical circuits become less integrative and more reactive, in line with a reduced low-pass filtering regime. Notably, the second eigenvalue exhibited a transient reversal of this trend around the response, suggesting a brief return toward a more integrative regime, in which the network temporarily accumulates variability rather than relaxing back to baseline—potentially reflecting a diffusion-like process associated with response monitoring (see *SI Appendix* for further discussion).

Third, emerging work on aperiodic EEG activity provides additional support. Developmental decreases in the spectral exponent and offset—two key aperiodic parameters—suggest a shift toward faster and less temporally correlated dynamics during adolescence (35–37). These spectral changes likely reflect underlying neurochemical processes. A recent longitudinal EEG-MRSI study (38) found that reductions in glutamate–GABA asymmetry in the prefrontal cortex—driven by decreases in glutamate—were associated with flatter spectral exponents. These findings align with the idea that synaptic pruning of excitatory inputs promotes more temporally concise neural activity, leading to decreased eigenvalues in our model.

Although pruning likely plays a central role, we acknowledge that it is not the only mechanism. Other concurrent processes—including increased myelination, changes in dopaminergic innervation, and inhibitory circuit refinement (39)—also shape cortical dynamics and likely interact with pruning to drive the observed developmental changes.

We next investigated whether childhood neglect alters the normative developmental trajectory of neural dynamics and found higher eigenvalues in participants who experienced institutional care relative to those raised in normative family environments. This suggests that early-life deprivation may disrupt or delay the normative developmental trajectory of neural circuit computations. This result partially contrasts with previous findings from the same cohort (40–42), where a recovery of some neural markers associated with error monitoring was observed following foster care intervention. Our approach differs from these previous findings in at least three fundamental ways. First, we focused on residual neural activity—the trial-by-trial deviations from the mean response—rather than on condition-averaged event-related potentials such as the ERN. This methodological approach allows us to assess recurrent computations within the network (43). By removing the average stimulus-locked input, our analysis emphasized the intrinsic dynamics of the system, which may be more sensitive to structural alterations in cortical circuitry than traditional ERP-based measures. Second, we employed principal component analysis to define a low-dimensional state space for modeling these dynamics. While this necessarily reduces spatial resolution—since each component reflects a linear combination of electrodes—it is essential for capturing population-level computations. In contrast, previous EEG studies in this cohort focused on a single electrode (typically FCGz) and on isolated ERP components. Third, and critically, our analysis was longitudinal: We tested the same individuals over time, modeling developmental trajectories of neural dynamics across ages 12, 16, and 21. This approach allowed us to account for individual developmental variability and provided a more direct assessment of how early-life adversity shapes the maturation of neural circuit dynamics.

Importantly, the neural findings paralleled the behavioral pattern, where evidence of recovery in the foster care group was modest and circumscribed. Further, the eigenvalues correlated with behavioral performance along expected directions, where faster decay to the mean linked to better performance (Fig. 5 *A* and *D*). Moreover, both the first and second eigenvalues partially mediated the relationship between age, institutionalization, and task performance, indicating that developmental and experiential effects on performance were, at least in part, transmitted through changes in neural dynamics (Fig. 5 *B*, *C*, *E* and *F*).

Within our theoretical framework, the observed disruptions of residual neural dynamics in institutionalized participants may reflect a delay or deviation in the normative trajectory of synaptic pruning and circuit stabilization in association cortices. This interpretation is consistent with neuroimaging findings showing widespread reductions in cortical thickness among previously institutionalized children compared to community controls, particularly in prefrontal, parietal, and temporal regions (44). While synaptic proliferation in the prefrontal cortex is maximal during the first years of life (45), pruning becomes most prominent during adolescence (4)—a developmental window that appears to be critically disrupted by early psychosocial deprivation (46). One proposed mechanism is that the lack of expected cognitive and social inputs from caregivers during sensitive periods alters the subsequent experience-dependent refinement, resulting in a developmental shift from the typical trajectory. Alternatively, it could represent accelerated pruning in response to deprivation (47).

In line with the theoretical framework here adopted, we favor the first hypothesis: that early deprivation disrupts the normative sequence of proliferation and pruning because of the reduced availability of expected experiential inputs during critical windows. This would lead to immature recurrent dynamics in adolescence, as indexed by elevated eigenvalues. However, this hypothesis remains to be directly tested in future animal-model studies combining longitudinal neurophysiological and anatomical data.

While this mechanistic framework offers a coherent account of our main findings, we also examined whether variability in placement stability and quality of the caregiving environment could moderate the developmental trajectory of neural dynamics, as these factors have been shown to associate with both biological maturation and cognitive–emotional functioning during adolescence (48, 49). Although neither variable showed a clear effect on the eigenvalue measures (*SI Appendix*, Fig. S3), the modest sample size likely limited statistical power to detect higher-order or interaction effects. Future studies with larger and more diverse samples should employ multivariate approaches to identify which factors modulate developmental trajectories of neural refinement, and under what conditions adaptive recovery can occur during adolescence.

To conclude, our findings provide evidence that adolescent maturation of neural dynamics—indexed by eigenvalue reductions—is altered by early-life deprivation, supporting the view that disrupted pruning trajectories underlie persistent differences in neural computation and behavioral performance.

## Materials and Methods

### Data Sample and Behavioral Analysis.

In the present study, we included a total of 163 participants recruited from the Bucharest Early Intervention Project (BEIP, see also *SI Appendix*, Fig. S4). The study was initiated at the request of the Secretary of State for Child Protection in Romania in collaboration with the Romanian Ministry of Health and has been approved by an ethics committee of Bucharest University and institutional review boards at the universities of the three principal investigators (C.A.N., C.H.Z., and N.A.F.). Written consent was obtained from each child’s legal guardian at each assessment timepoint, and verbal assent was obtained from young people beginning at the 12-y assessment timepoint.

Children under the age of 31 mo were assessed within three groups: Following random assignment, 52 children were moved from state-run orphanages to high quality foster care to become the Foster Care group (FCG); 51 children were randomized to remain in the orphanages as the Care As Usual group (CAUG); and 60 children, who were not randomized, were recruited from community settings and had no institutional exposure (never-institutionalized group [NIG]). Many children were directed to other placements over the course of the study by Romanian child protection authorities, and the investigators played no role in those decisions. Thus, the FCG represents children who experienced initial early deprivation, and CAUG represents children who experienced more sustained early deprivation. Data on these children were collected in follow-ups started at ages 12, 16, and 21 y (means ± SD: 12.66 ± 0.50; 16.86 ± 0.66; 23.34 ± 1.22).

All participants completed the Eriksen Flanker task, a well-established paradigm in developmental neuroscience for assessing selective attention and inhibitory control (26, 40). The task requires participants to respond to a target stimulus in the context of distracting stimuli. In this study, the target stimuli consisted of right- or left-facing arrows (Fig. 1*A*). Participants were instructed to respond as quickly and accurately as possible by pressing a button to indicate the direction of the middle arrow (right or left). Congruent trials contained stimuli all facing the same direction, whereas in incongruent trials, the central target was flanked by arrows facing the opposite direction. After a block of 20 practice trials, participants completed the experimental session, consisting of 160 test trials. Each trial began with a warning signal, followed by a blank screen, after which the flanker stimulus was presented (Fig. 1*A*). To maintain an overall accuracy of ~60%, we implemented a staircase procedure, adjusting the target presentation time based on participants’ cumulative accuracy: If accuracy exceeded 60%, the presentation time was reduced by 50 ms; otherwise, it was increased by 50 ms. Further details about the task are provided in ref. 50.

To examine whether task performance changed with age and was influenced by early adverse experiences, we used a linear mixed-effects model (LME) fit to three behavioral measures: accuracy (proportion of correct responses), reaction time (RT; the time between Flanker onset and button press), and the Balanced Integration Score (BIS). The BIS was computed as in ref. 51. By integrating standardized RT and accuracy measures with equal weighting, the BIS provides a composite index of performance efficiency that is largely insensitive to individual differences in speed–accuracy tradeoffs. We built the model according to the following formula:

[1] $$\begin{array}{c} Y=\beta_{0}+\beta_{1}Group+\beta_{2}Cond+\beta 3Age+\beta_{4}\left(Group\times Cond\right)\\ \;+\beta_{5}\left(Group\times Age\right)+\beta_{6}\left(Cond\times Age\right)+\beta_{7}\left(Group\times Cond\times Age\right)\\ \;+u_{0}+u_{1}Cond+u_{2}Age+u_{3}\left(Cond\times Age\right) \end{array}.$$

With *Y* being the dependent variable of interest. We included Group, Condition, and Age, as well as their two- and three-way interactions, as fixed effects. To account for individual differences in baseline performance, we included a random intercept for each participant (*u_0_* in the formula). Additionally, to model within-participant variability across ages and conditions, we specified random slopes for all within-participant variables (*u_1_*and *u_2_*in the formula) and their interactions (*u_3_* in Eq. **1**), allowing each participant to have their own trajectory across repeated measures. This structure allows the model to capture individual differences in performance trajectories while properly accounting for the nested design. Post hoc analyses were conducted by computing the appropriate contrast matrix to compare performance across specific levels of independent variables. Pairwise comparisons were corrected using the Bonferroni adjustment to control for Type I error. All F-statistics were calculated using the Satterthwaite approximation for degrees of freedom.

### EEG Recording and Analysis.

EEG data were recorded using a 64-channel HydroCel Geodesic Sensor Net and a NetAmps 300 amplifier (Electrical Geodesics, Inc., Eugene, OR). The signal was sampled at 500 Hz, with electrode impedances maintained below 50 kΩ. Data preprocessing followed a modified version of the MADE pipeline (52) adapted to the specifics of this study. The EEG data were first notch-filtered at 50 Hz to remove power line interference, followed by high-pass filtering at 1 Hz using a 4th-order IIR Butterworth filter to eliminate low-frequency drifts. A low-pass filter at 20 Hz (2nd order IIR Butterworth filter) was then applied to extract the evoked EEG activity, which is dominated by low-frequency oscillations. Eye-blink artifacts were detected and removed using independent component analysis (ICA) with the ADJUST toolbox (53). All data were then down-sampled to 40 Hz and segmented around two events of interest: from −1 to 1 s relative to the flanker stimulus and response onset, respectively.

Our goal was to apply dynamical systems analysis to investigate how age and early psychosocial deprivation shaped evoked-response dynamics. To this purpose, we analyzed the temporal evolution of neural state perturbations using methods previously applied to both spiking and EEG activity (9, 43). Fig. 1 summarizes the analysis pipeline. First, we z-transformed the EEG signal of each participant within each channel. Next, we computed the average Event-Related Potential (ERP) for each of the four conditions (congruent, incongruent, correct, and error, crossed) in each channel. The ERP was then time-concatenated, combining stimulus-locked and response-locked data. This resulted in an N (channels) × M (conditions × time points) matrix, which was then averaged across all participants and ages. We then computed the covariance of this matrix across channels, yielding an N × N covariance matrix, and applied Principal Component Analysis (PCA) to this matrix. Next, we projected all single-trial data for each participant into a subspace spanned by the first two PCs, which together accounted for more than 90% of explained variance. Within each participant, we then computed the condition average evoked potential for each trial type in each PC dimension. Trial-by-trial residual activity was obtained by subtracting the condition-averaged evoked activity (in the 2D PC space) from each single trial:

[2] $$x_{\mathrm{ij}}\left(t\right)=s_{\mathrm{ij}}\left(t\right)-{\bar{s}}_{j}\left(t\right),$$

Where *i* is a trial, *j* is a condition, and ${\overset{¯}{s}}_{j}$ is the trial average for that condition. This resulted in a 2D vector of residuals for each time point in each trial. For each participant, we then fit a linear dynamical systems (AR1) model to all the trials pooled together. To characterize the dynamical properties of residual activity, we used a time-varying 2D autoregressive model of the form:

[3] $$x\left(t+1\right)=A_{t}x\left(t\right)+\varepsilon \left(t\right),$$

where *A* is a 2 × 2 autoregressive coefficient matrix describing temporal dependencies in the residual activity ^′^*ε*, is the noise term and *x*(t) represents the residual activity in the PC space at time *t*. The model was fit to time-varying data, using a 200 ms moving time window (~25 ms step). We then extracted the eigenvalues of the matrix *A* at each time point. To investigate how age and early psychosocial deprivation impact the dynamics, we fit a LME to the eigenvalues:

[4] $$Eigenvalue=\beta_{0}+\beta_{1}Group+\beta_{2}Age+\beta_{3}\left(Group\times Age\right)+u_{0}+u_{1}Age.$$

As in the behavioral analysis, we included random intercepts and slopes to account for repeated measures, conducted post hoc analysis with Bonferroni correction, and approximated degrees of freedom for F-statistics using

Satterthwaite’s method. To study if eigenvalues were linked to behavioral performance (indexed by the BIS) and if such a relationship changes as a function of age or group, we fit a LME of the form

[5] $$\begin{array}{c} Eigenvalue=\beta_{0}+\beta_{1}BIS+\beta_{2}Age+\beta_{3}\left(Group\times Age\right)+\beta_{4}\left(BIS\times Age\right)\\ \;+\beta_{5}\left(BIS\times Group\right)+\beta_{6}\left(BIS\times Group\times Age\right)+u_{0}+u_{1}Age \end{array}.$$

To further test whether behavioral performance was mediated by eigenvalue dynamics, we conducted mediation analyses. Two models were estimated: one testing whether developmental changes in eigenvalues mediated the relationship between age and BIS, and another testing whether group differences (between never-institutionalized [NIG] and ever-institutionalized participants [FCG & CAUG]) in eigenvalues mediated the relationship between institutionalization and performance. For both models, eigenvalues were averaged within time windows that showed significant effects of age or group in the preceding LME analyses. Bootstrap resampling (5,000 iterations) was used to estimate path coefficients (a, b, c′) and the indirect effect (a × b), with bias-corrected and accelerated CI. In all time domain analyses, *P*-values were corrected via False Discovery Rate (FDR) to account for multiple comparisons over time. All analyses were performed using custom MATLAB (2023b) scripts and the Mediation Toolbox (https://github.com/canlab/MediationToolbox). This work used the computational resources of the NIH HPC Biowulf cluster (https://hpc.nih.gov/).

## Supplementary Material

Appendix 01 (PDF)

## Data Availability

Codes for data analysis and anonymized preprocessed data have been deposited in Zenodo (54).
