# Supplementary material for: Early psychosocial deprivation alters the refinement of neural dynamics across adolescence
Source: Proc Natl Acad Sci U S A. 2026 Feb 2;123(6):e2514979123. doi: 10.1073/pnas.2514979123 (PMC12890782; doi:10.1073/pnas.2514979123)
Supplement: Supplementary file 1 — Appendix 01 (PDF) [file pnas.2514979123.sapp.pdf]

Supplementary Materials

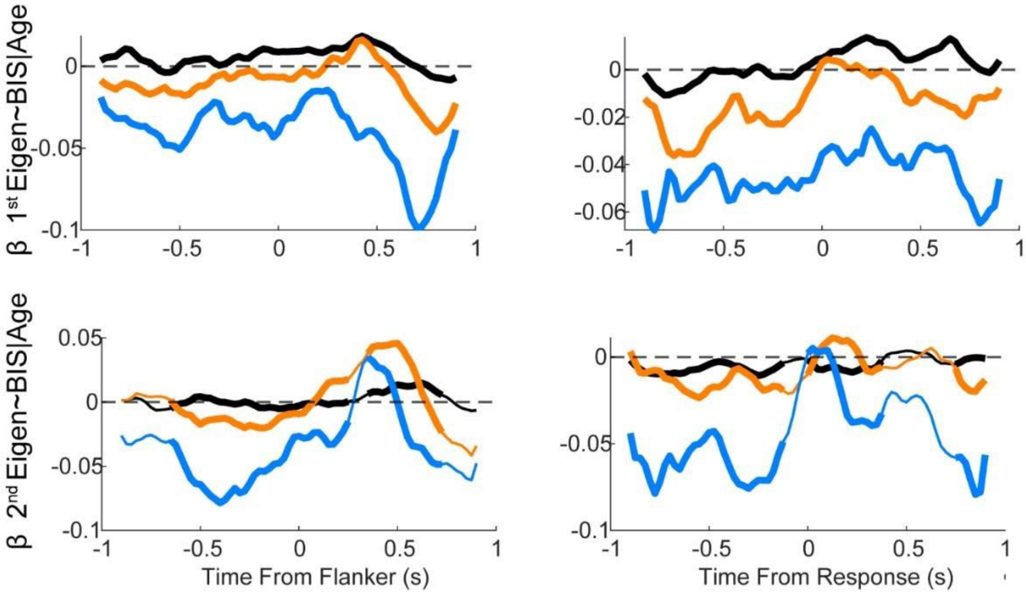

Supplementary Figure 1. Age-related modulation of the relationship between eigenvalue dynamics and BIS. Time-resolved β coefficients from linear mixed-effects models testing the association between eigenvalue magnitude and Balanced Integration Score (BIS), controlling for Age and Group (Eigenvalue ~ Age x Group x BIS + (Age |Subject)). Each line represents group-level trajectories for the 12-year-old (black), 16-year-old (orange), and 21-year-old (blue) participants. Top panels show results for the first eigenvalue aligned to the Flanker (left) and Response (right) onset. Bottom panels show the same effects for the second eigenvalue. Thick lines mark time points showing a significant Age × BIS interaction (p values FDRcorrected across time).

Supplementary Discussion – Transient neural integration during post-response monitoring

In our main analysis, we found that eigenvalues decreased with age across most task epochs, consistent with increasingly stable cortical dynamics. However, a notable exception emerged: immediately after the response, the second eigenvalue showed a transient rise in which the age effect inverted (Fig. 4B, Results).Given that this transient rise occurred immediately after the response—coinciding with the time window of performance monitoring—we sought to determine whether this effect was accompanied by behavioral and neural signatures of adaptive control. We examined post-error slowing (PES), a classic behavioral index of error monitoring and strategic adjustment. The PES was computed as the RT of correct post-error trials minus the RT of correct post-correct trials, following procedures commonly used in developmental studies of performance monitoring (1).

To study developmental and group differences, we used a linear mixed-effects model with random intercepts for participants (*PES ~ Age × Group + (1 + Age |Subject)*). This model tested both main effects of Age and Group, as well as their interaction, while accounting for repeated measures within subjects.

We found a significant main effect of Age ($F(2,137) = 5.12$, $p = 0.007$), but no significant effects of Group ($F(2,150) = 1.42$, $p = 0.24$) or Age × Group interaction ($F(4,136) = 0.10$, $p = 0.98$, Supplementary Fig.2A). These results indicate that the ability to adaptively modulate response speed after mistakes strengthens across adolescence, consistent with developmental improvements in performance monitoring and strategic control. We next focused on the neural correlates of this behavioral adjustment by examining response-locked activity associated with error detection and evaluation. Prior electrophysiological work has shown that the error-related negativity (ERN) is often preceded by a small positive deflection, the premovement positivity (PMP), emerging approximately 150–200 ms before the response over fronto-central sites. The PMP is thought to reflect a preparatory "go" signal generated in the pre-SMA and SMA, which facilitates response execution. Importantly, this component tends to be smaller before erroneous responses, suggesting that reduced pre-motor activation may precede action slips or failures of control (2).

Following the response, the ERN appears as a sharp negative deflection peaking around 100–150 ms, reflecting rapid error detection processes (3). This is often followed by a later error-related positivity (Pe), peaking between 200–400 ms, which indexes conscious awareness and evaluation of the error (4). Together, these components describe a temporal cascade of control-related processes— from proactive motor gating (PMP) to reactive error detection (ERN) and conscious evaluation (Pe).

To evaluate how these performance-monitoring components evolved across development, we computed the Δ(correct–error) response activity and projected it onto the first two principal components (PC1 and PC2), providing a low-dimensional summary of neural state changes during action monitoring. This approach allowed us to capture the main temporal modes of the response locked neural signal without relying on specific electrode locations or predefined ERP components.

The first principal component (PC1) encompassed the full sequence of control-related processes described above. It displayed an early positive deflection beginning ~150 ms before the response, consistent with the pre-movement positivity (PMP), followed by a sharp negative peak around 150 ms post-response (the error-related negativity, ERN) and a later positivity around 300 ms (the Pe). The second principal component (PC2) captured a later and more sustained post-response modulation, peaking between 200 and 300 ms, corresponding to a more evaluative stage of error processing.

Linear mixed-effects modeling of Δ(correct–error) amplitudes across age and groups (ΔERN ~ *Age × Group + (1 + Age |Subject)*) revealed significant developmental scaling in both components, with larger PMP, ERN, and Pe amplitudes observed in older participants (see Supplementary Fig.2B), while no significant Group or Age x Group effects were found. This indicates progressive strengthening of both proactive and reactive monitoring processes across adolescence.

When behavior is updated based on feedback, it requires integration of the outcome. More specifically, post-error slowing can be thought of us an adjustment of the speed-accuracy trade-off towards slower

responses that increase accuracy. From a dynamical perspective, integration of feedback is a process that requires a flat landscape. An attractor would resist change, because a feedback perturbation would fail to push activity out of the attractor. An integrator, on the other hand, would shift activity along a (possible) line-attractor in response to the feedback. Thus, integration of feedback should transiently reduce the contractive forces, flattening the local state-space landscape and giving rise to low-curvature directions along which the network state can evolve gradually in response to input (5). This reduction in local contraction should, in turn, be reflected in an increase of the corresponding eigenvalues toward unity, a pattern that we observed in the time window encompassing the ERN–Pe complex, and that is mirrored by behavioral adaptation (i.e. PES). Therefore, we propose that the apparent "reversal" of the age effect on the second eigenvalue does not reflect a breakdown of developmental stabilization, but rather a functional transition to a distinct operational phase of the system (i.e. integration).

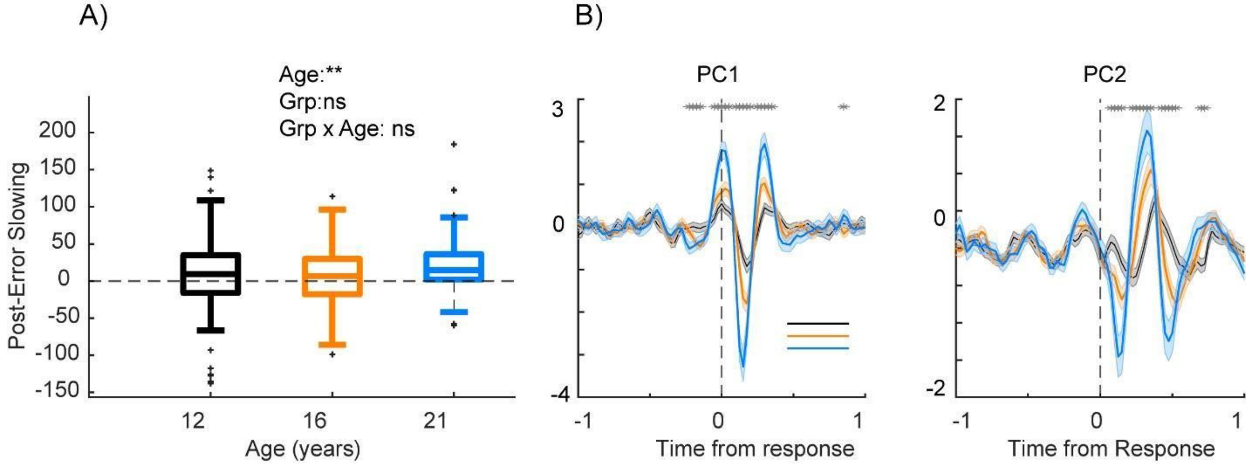

Supplementary Figure 2. Developmental modulation of post-error slowing and response-locked neural dynamics. (A) Post-error slowing (PES) across ages. Boxplots show median and interquartile range for the 12-year-old (black), 16-year-old (orange), and 21-year-old (blue) participants. Linear mixed-effects modeling revealed a significant main effect of Age ($p < 0.01$), but no significant effects of Group or Group × Age interaction. (B) Response-locked Δ(correct–error) activity projected onto the first two principal components (PC1, PC2). Shaded areas indicate ± SEM across participants. Asterisks mark time points showing a significant Age effect (FDR-corrected across time) same color code as in A).

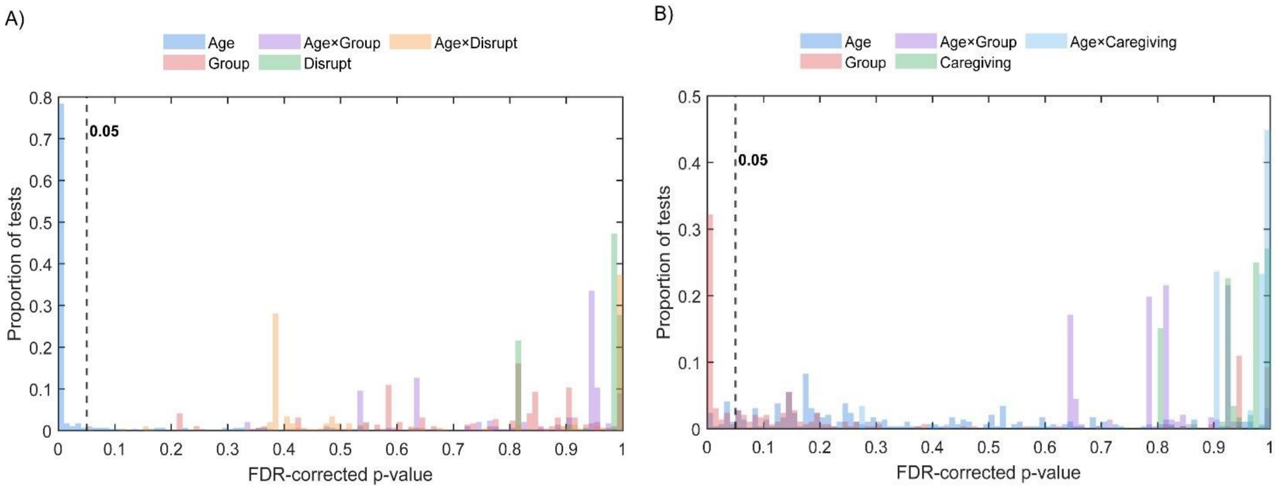

Supplementary Figure 3. A) Control analysis testing the effects of placement stability on eigenvalue dynamics. The histogram shows the distribution of FDR-corrected *p*-values across all time points and eigenvalues for each fixed effect in the mixed model (Eigenvalue ~ Age × Group + Disruptions + Age x Disruptions + (1 + Age | Subject)). Each color denotes a different main or interaction term: Age (blue), Group (red), Age × Group (purple), Disruptions (green), and Age × Disruptions (orange). The dashed vertical line indicates the 0.05 significance threshold. Only the main effect of Age yielded consistent clusters of significant effects, whereas all other terms—including those involving caregiving disruptions—remained non-significant after correction. Never-Institutionalized group (NIG) was excluded from this analysis, as disruptions were not defined for these participants. B) Same analysis as in panel A but controlling for *Caregiving quality*. The 21-year assessment was excluded because caregiving quality data were not available at that time point. The overall pattern of results remained unchanged, confirming that the developmental effects on eigenvalue dynamics were not accounted for by variability in the caregiving environment.

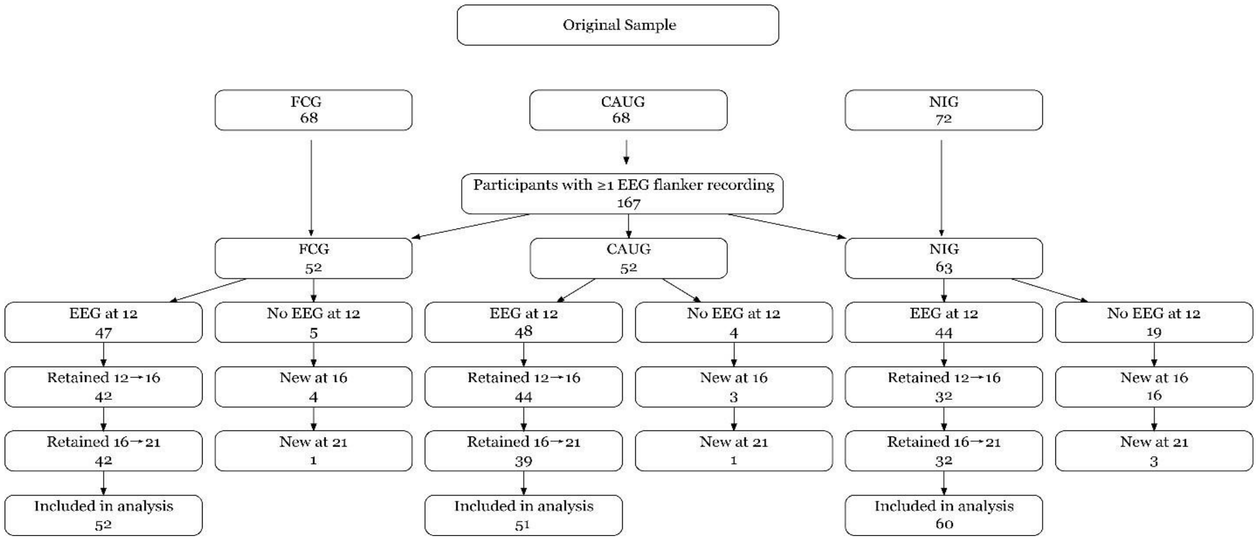

Supplementary Figure 4. The diagram summarizes participant retention and availability of usable EEG recordings across the three age waves (12, 16, and 21 years). Starting from the original sample (FCG = 68; CAUG = 68; NIG = 72), a total of 167 participants contributed at least one usable flankertask EEG recording. The figure shows, for each group, the number of participants with EEG data at age 12 and those missing at that wave, followed by the number *retained* or *newly entered* at ages 16 and 21 based on the presence or absence of EEG at the preceding timepoint. A total of 3 NIG participants and 1 CAUG participant were excluded due to poor-quality EEG recordings (more than 20% of trials were rejected, if more than 20% of electrodes required interpolation, or if PCA/ICA diagnostics indicated that less than 20% of the variance was accounted for by physiologically plausible components). The final analytical sample included 52 FCG, 51 CAUG, and 60 NIG individuals.

References:

1. E. Cravet, E. Ger, Exploring post-error slowing in children aged 8-11: task-specific patterns and associations with cognitive and academic outcomes. *J Exp Child Psychol* **261**, 106354 (2026).
2. M. Bortoletto, M. Sarlo, S. Poli, L. Stegagno, Pre-motion positivity during self-paced movements of finger and mouth. *Neuroreport* **17**, 883–886 (2006).
3. B. Albrecht *et al.*, Action monitoring in boys with attention-deficit/hyperactivity disorder, their nonaffected siblings, and normal control subjects: evidence for an endophenotype. *Biol Psychiatry* **64**, 615–625 (2008).
4. S. Nieuwenhuis, K. R. Ridderinkhof, J. Blom, G. P. Band, A. Kok, Error-related brain potentials are differentially related to awareness of response errors: evidence from an antisaccade task. *Psychophysiology* **38**, 752–760 (2001).
5. M. Khona, I. R. Fiete, Attractor and integrator networks in the brain. *Nat Rev Neurosci* **23**, 744–766 (2022).
